# Electroacupuncture Pretreatment Alleviates Myocardial Ischemia–Reperfusion Injury by Inhibiting Engulfment by Microglia in the Lateral Hypothalamus

**DOI:** 10.1111/cns.70595

**Published:** 2025-09-04

**Authors:** Xiang Zhou, Peiyi Yang, Chaonan Dong, Huimin Chang, Fan Zhang, Qi Shu, Naixuan Wei, Bin Zhang, Yan Wu, Wenjing Shao, Ronglin Cai, Qing Yu

**Affiliations:** ^1^ College of Traditional Chinese Medicine Anhui University of Chinese Medicine Hefei China; ^2^ Anhui Wannan Rehabilitation Hospital (The Fifth People's Hospital of Wuhu) Chinese Medicine Rehabilitation Department Wuhu Anhui Province China; ^3^ Medical College of Acu‐Moxi Anhui University of Chinese Medicine Hefei China; ^4^ Institute of Acupuncture and Moxibustion Meridian Anhui University of Chinese Medicine Hefei China; ^5^ Anhui Province Key Laboratory of Meridian Viscera Correlationship Anhui University of Chinese Medicine Hefei China; ^6^ Center for Xin'an Medicine and Modernization of Traditional Chinese Medicine Institute for Health and Medicine, Hefei Comprehensive National Science Center Hefei China

**Keywords:** electroacupuncture pretreatment, glutamate neuron, inhibitory synapses, lateral hypothalamus, microglia, myocardial ischemia–reperfusion injury

## Abstract

**Aim:**

The occurrence of myocardial ischemia–reperfusion injury (MIRI) is accompanied by neuroinflammatory reactions and is closely related to the overactivation of microglia. Currently, effective clinical strategies to prevent MIRI are unclear. This study investigated potential therapeutic targets and the mechanisms of electroacupuncture pretreatment (EA‐pre) for MIRI.

**Methods:**

A MIRI mouse model was established by ligating the left anterior descending branch of the heart for 30 min and reperfusion for 2 h. The mechanisms by which EA‐pre alleviates MIRI were investigated through immunofluorescence staining, chemogenetics, and fiber photometry recordings, focusing on the potential involvement of microglia and glutamate (Glu) neurons in the lateral hypothalamic (LH).

**Results:**

EA‐pre improves cardiac function in MIRI mice by suppressing microglial activation in the LH. The underlying mechanism likely involves EA‐pre inhibition of microglial engulfment of inhibitory synapses around LH^Glu^ neurons. Targeted activation of LH^microglia^ reverses EA's inhibitory effect, thereby increasing LH^Glu^ neuronal activity and triggering overactivation of the sympathetic nervous system (SNS), which ultimately exacerbates MIRI.

**Conclusion:**

EA‐pre inhibits microglial engulfment of inhibitory synapses around LH^Glu^ neurons in MIRI mice, thereby suppressing LH^Glu^ neuronal activity, reducing SNS output, and ultimately exerting cardioprotective effects.

## Graphical abstract

During MIRI, enhanced microglial engulfment of inhibitory synapses around LH^Glu^ neurons leads to increased LH^Glu^ neuronal activity. This will exacerbate myocardial damage. EA‐pre improves cardiac function by suppressing microglial activation in the LH. The underlying mechanism likely involves EA‐pre mediated inhibition of microglial engulfment of inhibitory synapses around LH^Glu^ neurons, which suppresses LH^Glu^ neuronal hyperactivity, reduces SNS output, and ultimately protects cardiac function.

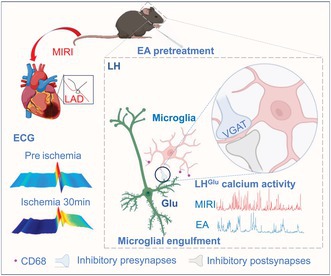

## 1 Introduction

Ischaemic heart disease is a common and serious cardiovascular disease with high morbidity and mortality rates [1, 2]. At present, timely and effective vascular reconstruction techniques can effectively restore myocardial blood supply, alleviate myocardial injury, and improve patients' quality of life [3]. However, further exacerbation of cell and tissue damage during reperfusion remains one of the clinical challenges [4]. Pretreatment is considered to be an effective way to improve myocardial ischemia–reperfusion injury (MIRI); therefore, exploring safe and effective treatments has become a hot research topic in this field [5]. Acupuncture can regulate the autonomic nervous system (ANS) [6, 7], promote physiological balance, and has been widely recognized in the treatment of cardiovascular diseases [8]. Clinical studies have shown that electroacupuncture pretreatment (EA‐pre) can alleviate perioperative myocardial injury after interventional procedures, especially in patients with complex coronary artery lesions [9].

MIRI not only causes myocardial injury, but also damages other organs including the brain, which is closely related to the poor clinical prognosis of myocardial ischemia [10, 11]. The inflammatory signals it triggers are transmitted to the dorsal vagal complex of the brainstem through different structures and further project to the brainstem and brain regions [12]. The brain is composed of various types of cells, most of which come from neural stem cells in the central nervous system (CNS) [13]. Microglia, as key immune cells in the CNS, originate from primitive macrophages generated in the yolk sac during embryonic development [14]. In the CNS, microglia not only have the ability to proliferate, but also participate in maintaining the homeostasis of the nervous system [15]. They differ significantly in phenotype from macrophages in other tissues, suggesting that they have unique biological properties, and play a crucial role in the brain's immune defense system [16, 17]. The morphological state of microglia directly reflects their functional diversity [18]. Numerous studies have shown that in ischemic diseases, microglia not only recognize and engulf synapses through specific phagocytic receptors, but also regulate synaptic plasticity by releasing various effector molecules [19]. Following myocardial infarction, microglial activation in the hypothalamus has been observed [20]. This leads to elevated pro‐inflammatory cytokine levels that activate the hypothalamic–pituitary–adrenal axis, increase sympathetic nervous system (SNS) activity, and contribute to acute cardiac inflammation post‐infarction [21]. In addition, increasing evidence suggests that microglia actively participate in shaping and regulating neuronal activity by forming dendritic spines and regulating the distribution of neurotransmitter receptors at synaptic terminals [22].

In summary, whether LH^microglia^ are involved in the mechanism of action of EA to attenuate MIRI is unclear, and relevant studies are lacking. Our previous research has shown that EA mediates the impact of LH^Glu^ neurons on SNS and plays a key role in MIRI [23]. However, whether microglia are involved in the regulatory mechanism of EA on LH^Glu^ neurons has not been clearly confirmed. Therefore, this study aims to combine immunofluorescence staining, Western blot (WB), chemogenetic, in vivo multi‐channel electrophysiological recording, and fiber photometry recordings to investigate the mechanism of EA‐pre at the HT7 acupoints in modulating engulfment by LH^microglia^ to alleviate MIRI, and further elucidate its role in improving cardiac function by affecting the sympathetic nervous system through LH^Glu^ neurons.

## 2 Materials and Methods

### 2.1 Animals

The experimental mice were male C57BL/6 mice (20–25 g, 8w), purchased from Hangzhou Ziyuan Experimental Animal Science and Technology Co. Ltd. (license number: SCXK (Zhe) 2019‐0004), and raised in the Animal Laboratory of Acupuncture and Moxibustion Meridian Research Institute of Anhui University of Traditional Chinese Medicine. During the experiment, an independent ventilated mouse cage (Monkey King HH‐A‐4 II type, Suzhou, China) was used, with a feeding temperature maintained at 22°C–24°C and humidity controlled within the range of 50%–60%. Mice can obtain water and food at any time, and natural light alternates between light and dark for 12 h. This experiment has been approved by the Animal Experiment Ethics Committee of Anhui University of Traditional Chinese Medicine (Approval Number: AHUCM‐muse‐2022083).

### 2.2 Establishment of MIRI Model

Mice were anesthetized with 1.5% isoflurane (1 L/min) and fixed in the supine position on the operating table. Connect the electrodes and record the electrocardiogram (ECG). Separate the chest layer by layer, expose the heart, and ligate the left anterior descending coronary artery using non‐invasive sutures. After 30 min, release the ligature and close the chest cavity for 120 min of reperfusion. If the S‐T segment deviation drops by more than 50% after loosening the ligature and restoring blood supply, it indicates that the MIRI model has been successfully established.

### 2.3 Electroacupuncture Pretreatment

Bilateral HT7 acupoints were needled using disposable sterile needles (0.25 mm × 13 mm, Jiangsu Tianxie Acquisition Instrument Co. Ltd., Jiangsu, China). HT7 is located approximately 1–22 mm above the wrist joint on the ulnar side of the mouse wrist and is connected to the positive electrode. The EA at the nonacupoint (EA NA) group is treated by inserting needles into the tail of mice. Acupuncture depth is about 3 mm, connected to an EA device (HANS‐200A, Jisheng Medical Technology Co. Ltd., Nanjing, China), using a 2 Hz continuous wave with a current intensity set at 1 mA, for 20 min, once a day, for 7 days of treatment.

### 2.4 ECG Recording

ECG signals are acquired through Standard II leads of PowerLab. The LabChart8 software is employed to analyze the S‐T segment deviation values and the low‐frequency/high‐frequency (LF/HF) ratio. The measurements are taken at three time points: 5 min prior to ischemia, 30 min following myocardial ischemia, and 120 min after reperfusion (low‐cut filter, 200 Hz; high‐cut filter, 0.3 Hz) [24]. Continuous recording lasts for 5 min, and the average of each minute is computed. Subsequently, the LF/HF ratio is calculated based on these data.

### 2.5 Triphenyl‐Tetrazolium Chloride‐Evans Blue Double Staining

The heart was surgically exposed, and 0.1 mL of 0.5% Evans Blue (Leagene, Anhui, China) was injected into the cardiac apex. Subsequently, the heart was frozen, and then vertically sliced along the long axis beneath the ligation site, with an approximate thickness of 0.1 mm. The slices were soaked in a 2% TTC solution (Solarbio, Beijing, China) and incubated at 37°C for 15 min in the absence of light. Image J was utilized to perform imaging for the analysis of the area at risk (AAR) and infarction area (IA). The uninjured myocardium was stained blue, the IA was uncolored, and the AAR was stained red.

### 2.6 Echocardiography

The coupling agents were evenly applied to the left side of the mouse sternum, and a clinical small animal ultrasound imaging system (Vinno 6 Lab, China) equipped with a 23 MHz probe was utilized to detect left ventricular function [25]. The left ventricular ejection fraction (EF) and fractional shortening (FS) of the heart were evaluated, and the average of measurements obtained from three consecutive pulsation cycles was analyzed.

### 2.7 Immunofluorescence Staining and Analysis

Brain tissue was sectioned under freezing conditions with a thickness of 60 μm. The brain slices were then cleaned and placed in a containment solution consisting of 0.5% Triton X‐100 and 3% BSA. Subsequently, the primary antibody was added dropwise, and the slices were incubated overnight at 4°C. The primary antibodies used included: rabbit anti‐Iba‐1 (1:1000, Woka, 019‐19741), pig anti‐Iba‐1 (1:400, SYSY, 234308), rat anti‐CD68 (1:50, Abd Serotec, MCA1957GA), rabbit anti‐c‐Fos (1:1000, SYSY, 226008) and rat anti‐VGAT (1:1000, SYSY, 131002). On the following day, the brain slices were flipped and washed three times with PBS, with each wash lasting for 5 min. Subsequently, fluorescently labeled secondary antibodies were added dropwise and incubated at room temperature for 2 h under light‐avoidance conditions. The secondary antibodies employed were Goat Anti‐Rabbit IgG 594 (abcam, ab150080), Goat Anti‐Rabbit IgG 488 (abcam, ab150077), Goat Anti‐Rat IgG 647 (abcam, ab150167) and Donkey Anti‐Pig 647 (SYSY, 706‐605‐148). The brain slices were again washed three times with PBS, each wash lasting 5 min. Finally, the brain slices were transferred to slides with drops of DAPI sealing solution.

### 2.8 Western Blot

Mouse brain tissue was rapidly removed on ice and placed into a mouse brain mold (0–75 g, coronal sections; Shanghai Yuyan Scientific Instrument Co. Ltd.) for LH isolation. The cell lysate was subjected to centrifugation, and the supernatant was collected. Subsequently, electrophoresis, membrane transfer, and membrane sealing were carried out in sequence. The primary antibody was added dropwise, and the sample was incubated at 4°C overnight. After washing the membrane, it was incubated with the secondary antibody (diluted at a ratio of 1:20,000) for 1.5 h. Finally, the membrane was washed again, and protein expression was detected following the standard protocol.

### 2.9 Viral Injection

The skull plane was adjusted to a horizontal position, and holes were drilled on both sides above the injection site (LH: AP: −1.05 mm, ML: ±1.1 mm, DV: −5.10 mm). The glass microelectrode syringe pump was configured to inject at a rate of 40 nL/min, with an injection volume of 100 nL per side. Upon completion of the injection, the pipette was left in place for 15 min to facilitate virus spread and transfection. Finally, the scalp was sutured and sterilized.

### 2.10 In Vivo Electrophysiological Recordings

The skull plane was adjusted to a horizontal position. On the cranial surface above one side of the LH, a cranial window with a size of approximately 2 × 2 mm was cut using a cranial drill, and an 8‐channel microfilament electrode was positioned above the LH. The electrode tip was slowly advanced towards the LH at a speed of 10 μm/s. Once stable neuronal firing was observed, the signals were recorded for a duration of 5 min. Signal acquisition was carried out using a Plexon in vivo multi‐channel signal acquisition system (OPX010, USA), and the acquired signals were analyzed by employing NeuroExplorer 5.

### 2.11 Three‐Dimensional Reconstruction

Imaging was performed using an Olympus FV31S microscope. For all imaging experiments, consistent imaging parameters, namely gain, offset, and filter settings, were utilized. A particular region within the LH was selected for microglia imaging. *Z*‐axis superimposed images were acquired with a step size of 1 μm. Subsequently, 1024 × 1024‐pixel images were reconstructed using IMARIS 10.2.0 software (BitPlane).

IMARIS software was employed to conduct three—dimensional (3D) surface reconstruction and analysis of microglia. The “filament” function within IMARIS was utilized to measure the number of branch points and the length of microglial protrusions. Then use the plugin ‘Split into Surface Objects’ to evaluate the number of VGAT^+^ spots around neurons and microglia cells separately. For analysis of contact between microglial processes and neuronal dendrites contacts, Iba‐1^+^ microglia and mGFP^+^ dendrites were reconstructed using the “Surface” function in Imaris software. Then the plugin “Surface‐Surface Contact Area” was applied to assess the size of contact areas between microglial processes and neuronal dendrites.

### 2.12 Optical Fiber Implantation and Fiber Photometry Recordings

The rAAV‐vglut2‐GCaMP6s‐WPRE‐hGH polyA (SHUMI, PT‐3722) virus was injected into the LH. A black ceramic inserted optical fiber was vertically implanted 0.2 mm above the viral injection site using an optical fiber gripper. Subsequently, an instantaneous adhesive was applied to cover the cranial surface of the mice, and dental cement was used to secure the coverage.

After the mice underwent the operation, they were housed in a container with adequate space. After the mice were naturally awakened, the fiber‐optic connecting wire was fixed to the head of the mice to ensure that they could move freely. Recordings were performed using a DualColorMultichannel Fiber Optic Recording System (m17498, Qianaoxingke, Nanjing, China), which stimulated the target neuronal nucleus through the fiber optic to monitor its calcium signaling activity, and the light intensity was set at 0.03 mW. Finally, the DualColorMultichannel TP_405_470 (Qianaoxingke, Nanjing, China) acquisition software was used at a frequency of 100 Hz to record neuronal activity in vivo. Data analyses were processed using Matlab MAT software.

### 2.13 Statistical Analyses

All experimental data were analyzed using GraphPad Prism 8 software, with results presented as mean ± standard deviation (SD). All data were evaluated for normality using Bartlett's test and Brown‐Forsythe test. Data conforming to normal distribution were analyzed by one‐way ANOVA with 95% confidence intervals, followed by Tukey's post hoc test for multiple comparisons. Non‐normally distributed data underwent nonparametric tests. Statistical significance was defined as *p* < 0.05.

## 3 Results

### 3.1 EA‐Pre at HT7 Acupoints Protects Cardiac Function

**FIGURE 1:**
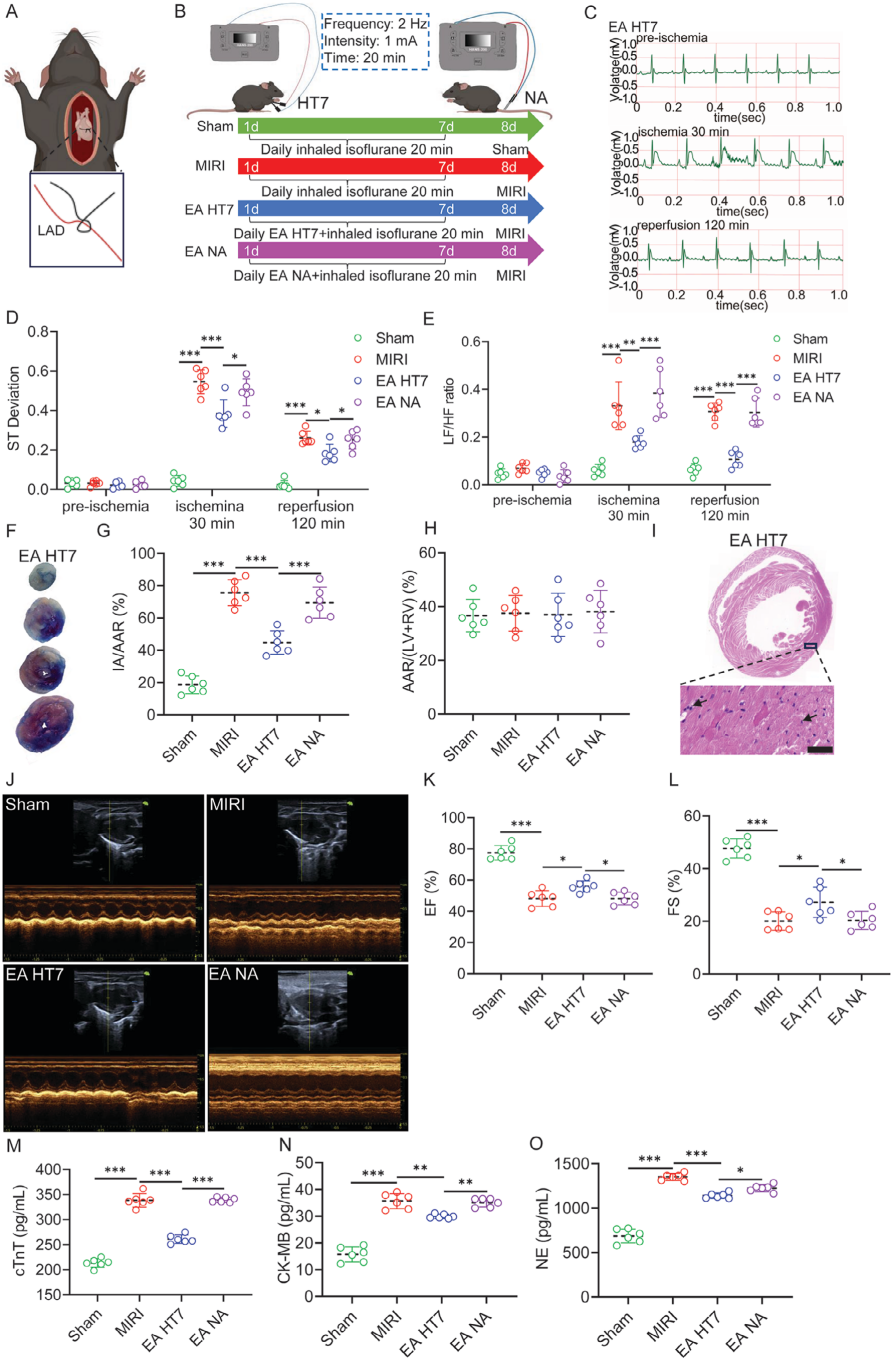
EA‐pre effectively protects cardiac function. (A) Open chest ligation of the left anterior descending branch of the coronary artery in mice. (B) Schematic diagram of EA treatment. (C) The ECG recording with EA‐pre at HT7 of MIRI mice. (D) A comparison of S‐T segment deviation in each group of mice. (E) A comparison of the LF/HF ratio in each group. (F) The photos of Evans blue‐TTC double staining. (G–H) A comparison of the percentage of IA and AAR in each group of mice. (I) Hematoxylin and eosin staining showing the morphology of myocardium in each group. Scale bar = 100 μm. (J) Left ventricular echocardiography in each group. (K,L) A comparison of EF and FS values of left ventricular echocardiography in each group. (M‐O) The statistical analysis of cTnT, NE and CK‐MB concentrations of left ventricular myocardial tissue homogenates in each group. All data are represented by one‐way ANOVA with Tukey's post‐test, *n* = 6 mice per group. *p < 0.05, **p < 0.01 and ***p < 0.001.

We established a MIRI model by opening the chest and ligating the left anterior descending artery of the heart (Figure 1A), and selected bilateral HT7 acupoints as EA stimulation sites (Figure 1B). In addition, to investigate the specificity of acupoints, we performed non‐acupoint electrical stimulation on the tail of mice to exclude the effects of electrical and physical stimulation (Figure 1B). ECG recordings of cardiac activity were performed in all groups to reflect the degree of impairment of MIRI (Figures 1C and S1A). We observed that the S‐T segment was significantly elevated in MIRI mice, while EA decreased the S‐T segment (Figure 1D). This suggests that EA can attenuate MIRI. It is well known that cardiac functional activity is regulated by the ANS [26], and heart rate variability (HRV) is an important index for evaluating the function of the ANS [27]. Therefore, we investigated the low frequency/high frequency (LF/HF) ratio, an important indicator reflecting HRV. It can be assumed that power in different frequency bands corresponds to sympathetic (0.04–0.15 Hz) and parasympathetic (0.15–0.4 Hz) activity [28]. The results showed that HRV was significantly elevated in MIRI mice, whereas EA suppressed the abnormal elevation of HRV and corrected the sympathetic–parasympathetic balance (Figure 1E). Interestingly, EA at non‐acupoints cannot alleviate the S‐T segment and LF/HF ratio, which precisely reflects the specificity of acupoints.

We also observed the degree of myocardial ischemia in each group of mice (Figures 1F and S1B). The results showed that the IA of MIRI mice significantly increased, while only EA‐pre at HT7 acupoints reduced IA (Figure 1G). Interestingly, there was no statistically significant difference in AAR among the groups (Figure 1H). HE staining of myocardial tissue similarly revealed increased myocardial inflammatory infiltration and injury in MIRI mice. EA‐pre HT7 reduced inflammatory infiltration and attenuated myocardial injury to some extent (Figures 1I and S1C).

In addition, the EF and FS of MIRI mice decreased, while EA increased EF and FS, protecting cardiac function (Figure 1J–L). Similarly, an increase in Cardiac Troponin T (cTnT) and Creatine Kinase‐Myocardial Band (CK‐MB) concentrations in MIRI mice indicates myocardial injury, while an increase in norepinephrine (NE) concentration indicates abnormal SNS excitation. However, EA‐pre HT7 reversed the aforementioned phenomenon (Figure 1M–O). The results of serum cTnT, IL‐1β, and IL‐6 detection also indicate that EA HT7 has an improving effect on myocardial injury and inflammatory response (Figure S1D–F). The above results indicate that EA‐pre at HT7 acupoints can effectively alleviate MIRI, protect cardiac function, and the HT7 acupoint plays a crucial role in it.

### 3.2 EA‐Pre Inhibits the Engulfment by Microglia in LH and Alleviate MIRI

**FIGURE 2:**
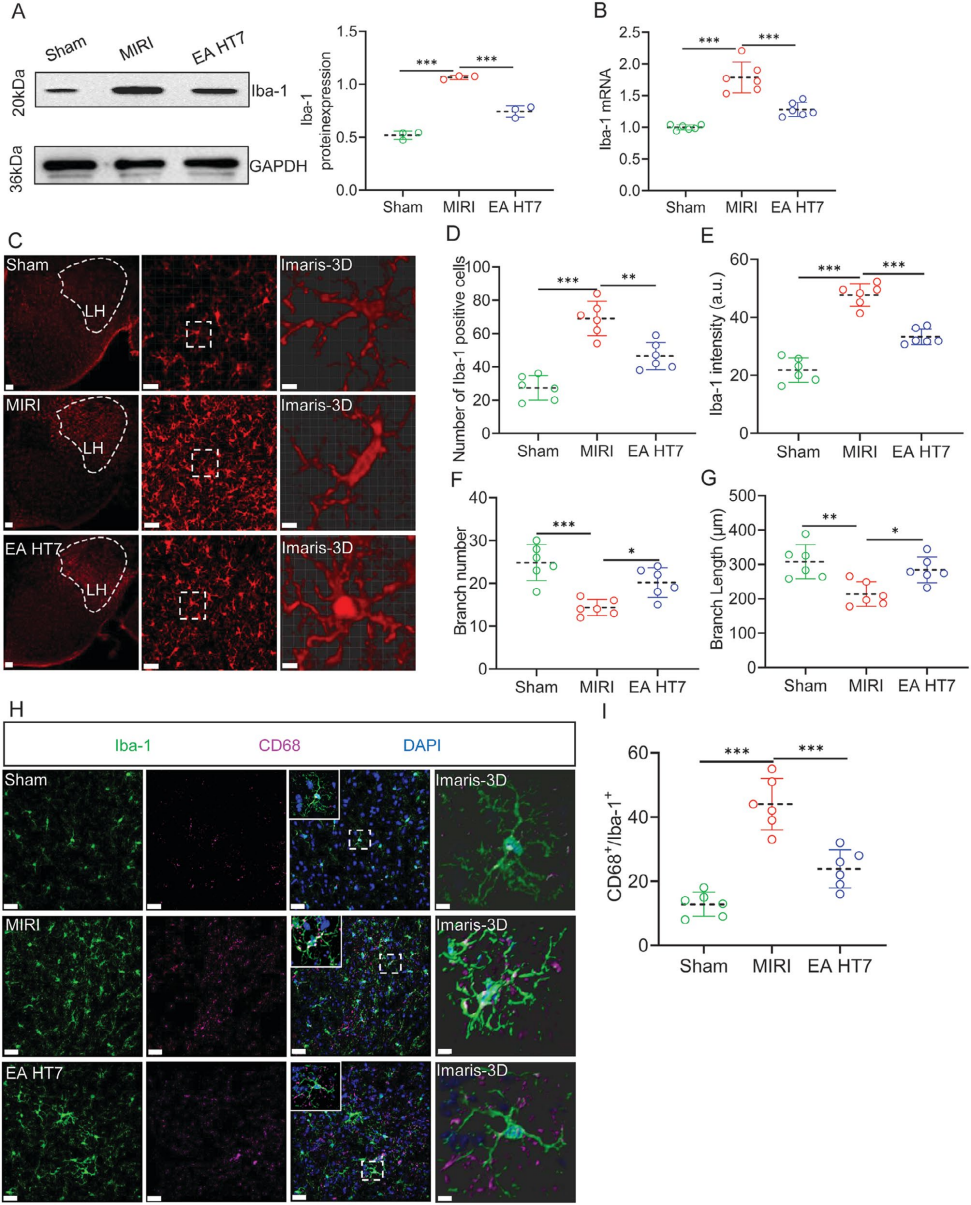
LH^microglia^ are involved in EA‐pre to alleviate MIRI. (A) LH expression levels of Iba‐1^+^ in each group of mice (one‐way ANOVA with Tukey's post‐test, ***p < 0.001, *n* = 3 per group). (B) A comparison of Iba‐1 mRNA expression in each group of mice (one‐way ANOVA with Tukey's post‐test, ***p < 0.001, *n* = 6 per group). (C) Images show 3D reconstruction of LH^microglia^ (red) in Sham group, MIRI group, and EA HT7 group. Scale bar = 400 μm (left); Scale bar = 20 μm (middle); Scale bar =10 μm (right). (D‐G) The number of Iba‐1^+^ cells per 0.1 mm^2^, Iba‐1^+^ intensity, branch number and branch length of Iba‐1^+^ in the LH from the mice in the Sham, MIRI, and EA HT7 groups (one‐way ANOVA with Tukey's post‐test, ***p < 0.001, **p < 0.01, *p < 0.05, *n* = 6 mice per group) (H) Representative image of CD68^+^ (purple) expression in Iba‐1^+^ (green) cells in the LH. Scale bar = 20 μm; Scale bar =10 μm (Imaris‐3D). (I) Analysis of the proportion of CD68^+^ in Iba‐1^+^ cells (one‐way ANOVA with Tukey's post‐test, ***p < 0.001, *n* = 6 per group).

During MIRI, excessive activation of microglia is closely related to brain cell apoptosis and neuroinflammation [29]. Therefore, we examined ionized calcium‐binding adaptor molecule 1 (Iba‐1) in the LH, which is a microglia‐specific expressed protein (Figure 2A,B). We found that EA inhibited the overexpression of microglia caused by MIRI. In addition, we performed 3D reconstruction of microglia (Figure 2C). It was also found that EA‐pre suppressed the number of Iba‐1^+^ cells, fluorescence intensity, number of branches, and increased branch length compared to the MIRI group (Figure 2D–G). Microglia are activated as immune effector cells and prune synapses by their own phenotype [30]. Thus, we observed that CD68^+^, which reflects the phagocytic ability of microglia, significantly increased during MIRI, and EA inhibited this phenomenon (Figure 2H,I). The above research indicates that during MIRI, EA‐pre inhibits the engulfment by LH^microglia^.

### 3.3 Microglia Mediate the Involvement of LH Neurons in MIRI

**FIGURE 3:**
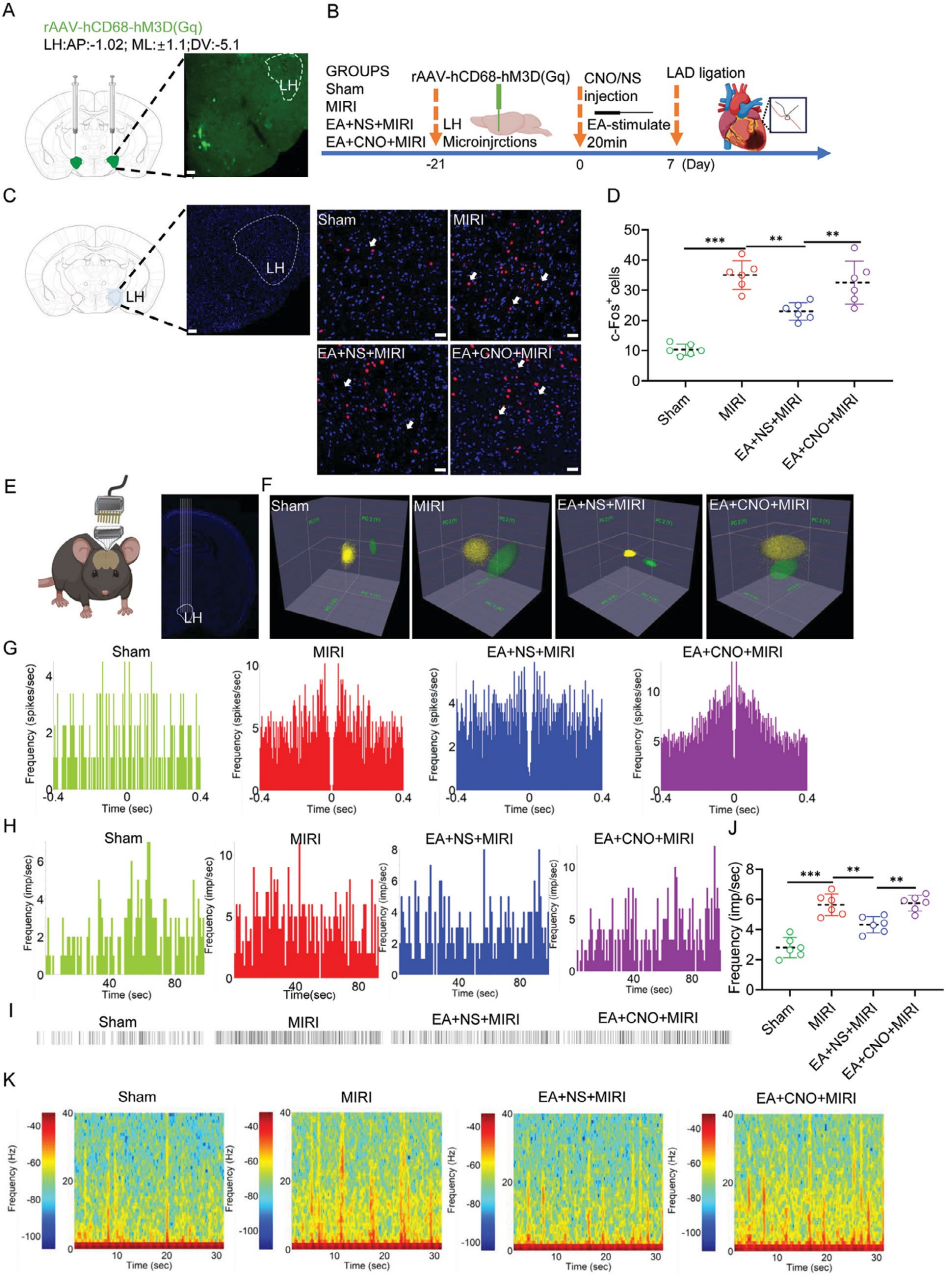
Microglia regulate the electrical activity of LH neurons during MIRI. (A) Virus injection location. Scale bar = 400 μm. (B) Schematic diagram of virus regulation. (C) Representative images of the c‐Fos^+^ cells in the LH. Scale bar = 100 μm. (D) The statistical analysis of the number of c‐Fos^+^ cells in each group. (E) Electrode implantation schematic and morphological verification of LH electrode location. (F) Autocorrelation analysis of neuronal firing sequences. (G) Autocorrelation diagram of excitatory neurons' electrical activity. (H) Tracking of excitatory neurons firing frequencies in the LH. (I) Raster images of excitatory neurons electrical activity in the LH. (J) Comparison of excitatory neurons firing frequencies in each group. (K) Comparison of spectral energy diagrams among the groups (time = 30 s, frequency = 40 Hz). All data are represented by one‐way ANOVA with Tukey's post‐test, *n* = 6 mice per group. **p < 0.01 and ***p < 0.001.

To further elucidate the mechanism by which LH^microglia^ mediate the protective effects of EA‐pre to alleviate MIRI, we designed a virus (rAAV‐hCD68‐hM3D(Gq); SHUMI, PT‐3768), which can target and activate microglia (Figure 3A). We performed immunofluorescence staining on microglia (Figure S2A) to validate their expression efficiency (Figure S2B). After virus expression, clozapine N‐oxide (CNO) was injected intraperitoneally to regulate the activity of microglia (Figure 3B). Similarly, we also detected the protein expression level and gene detection of Iba‐1 in LH (Figure S3C,D), confirming that EA‐pre mediates LH^microglia^.

Previous studies have shown that LH^Glu^ is involved in EA to alleviate MIRI. Therefore, to investigate the relationship between microglia and neurons, we performed c‐Fos^+^ cells immunofluorescence staining in the LH (Figure 3C). Surprisingly, the activation of LH^microglia^ reversed the inhibitory effect of EA on c‐Fos^+^ cells in LH, indicating that EA may alleviate MIRI by regulating LH^Glu^ through microglia (Figure 3D).

In order to clarify which types of neurons are specifically associated with LH^microglia^, we embedded multi‐channel electrodes in the LH (Figure 3E). Two types of neuronal activity were detected within the LH (Figure 3F). Autocorrelation analysis was performed on these two types of neurons detected as excitatory neurons (Figure 3G) and inhibitory neurons (Figure S2E) [31]. We found that the firing frequency of excitatory neurons in the LH of MIRI mice was significantly increased, and the activation of microglia weakened the inhibitory effect of EA on the excitatory neurons in LH (Figure 3H–J). Interestingly, the activation of microglia has no effect on the firing frequency of inhibitory neurons (Figure S2F–H). Next, we detected the cluster activity of neurons in LH, among which the MIRI group had the strongest potential energy spectrum, while the activation of microglia weakened the inhibitory effect of EA on the potential energy spectrum (Figure 3K). The above results suggest that microglia activation may attenuate the inhibitory effect of EA‐pre on excitatory neurons in the LH, which provides positive evidence that microglia mediate the involvement of LH^Glu^ neurons in MIRI.

### 3.4 EA‐Pre Inhibition of Microglia Engulfment of VGAT Results in Inhibition of LH^Glu^ Neurons

**FIGURE 4:**
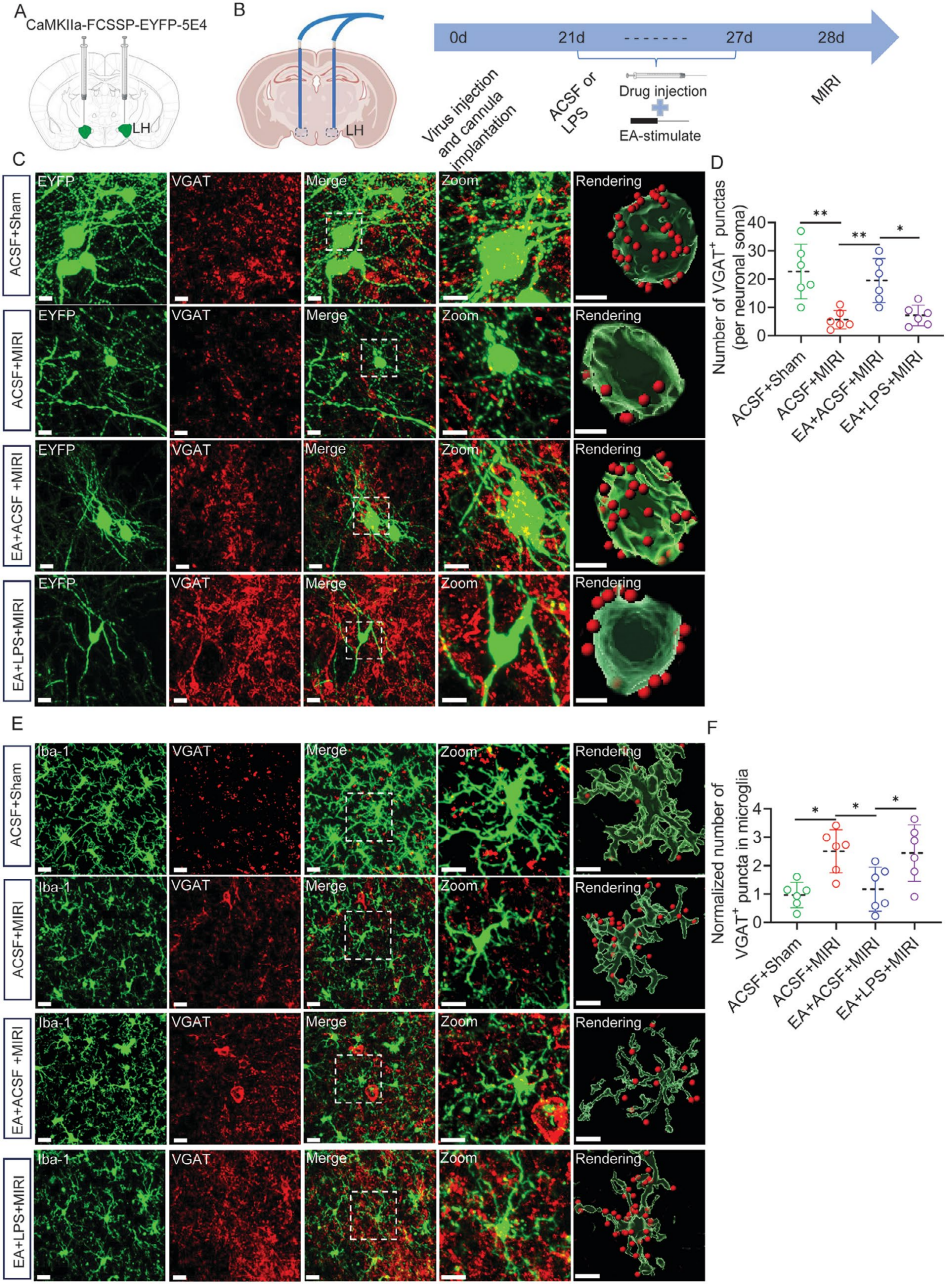
EA‐pre inhibits microglial engulfment of inhibitory synapses. (A) Strategy map showing sparse labeling of LH^Glu^ neurons. (B) Schematic diagram of cannula implantation in the LH. (C) Images of 3D rendering of VGAT (red) and EYFP (green) in the LH in each group of mice. Scale bar = 20 μm (overview) or 5 μm (inset) and 10 μm (rendering). (D) Quantification of VGAT^+^ puncta on the surface of EYFP^+^ neuronal soma in each group of mice. (E) Images of 3D rendering of VGAT (red) and Iba‐1 (green) in the LH in each group of mice. Scale bar = 20 μm (overview) or 10 μm (inset and rendering). (F) Quantification of VGAT+ puncta in microglia in the LH in each group of mice (one‐way ANOVA with Tukey's post‐test, **p < 0.01 and *p < 0.05, *n* = 6 mice per group).

Since neuronal activity depends on the balance between excitatory and inhibitory inputs, we also investigated whether changes in inhibitory presynapse around LH^Glu^ neurons in MIRI mice lead to increased LH^Glu^ activity. We injected CaMKIIa‐FCSP‐EYFP‐5E4 (brainvta, BC‐SL014) into LH to sparsely label LH^Glu^ neurons (Figures 4A and S3A) and implanted a cannula (Figure 4B). After 21 days lipopolysaccharide (LPS) was injected into LH before EA every day and compared with artificial cerebrospinal fluid (ACSF). Immunofluorescent detection of the presynaptic component marker, vesicular γ‐aminobutyric acid transporter (VGAT) (Figure 4C), showed that the number of VGAT^+^ puncta significantly decreased on EYFP^+^ neuronal soma in the LH of MIRI mice (Figure 4D). Notably, EA‐pre attenuated this synaptic loss, while microglial activation weakened this inhibitory effect of EA. This result indicates that the inhibitory input to LH^Glu^ neurons in MIRI mice is reduced, while EA can increase the inhibitory input to Glu neurons. As is well known, microglia interact with neurons and can modulate neuronal activity through synaptic pruning. 3D reconstruction showed an increase in co‐localization between microglia and VGAT during MIRI, while EA reduced the co‐localization spots in LH. Activation of microglia weakened this effect (Figure 4E,F). The above results indicate that EA‐pre may inhibit microglial engulfment of inhibitory synapses around LH^Glu^ neurons in MIRI mice.

**FIGURE 5:**
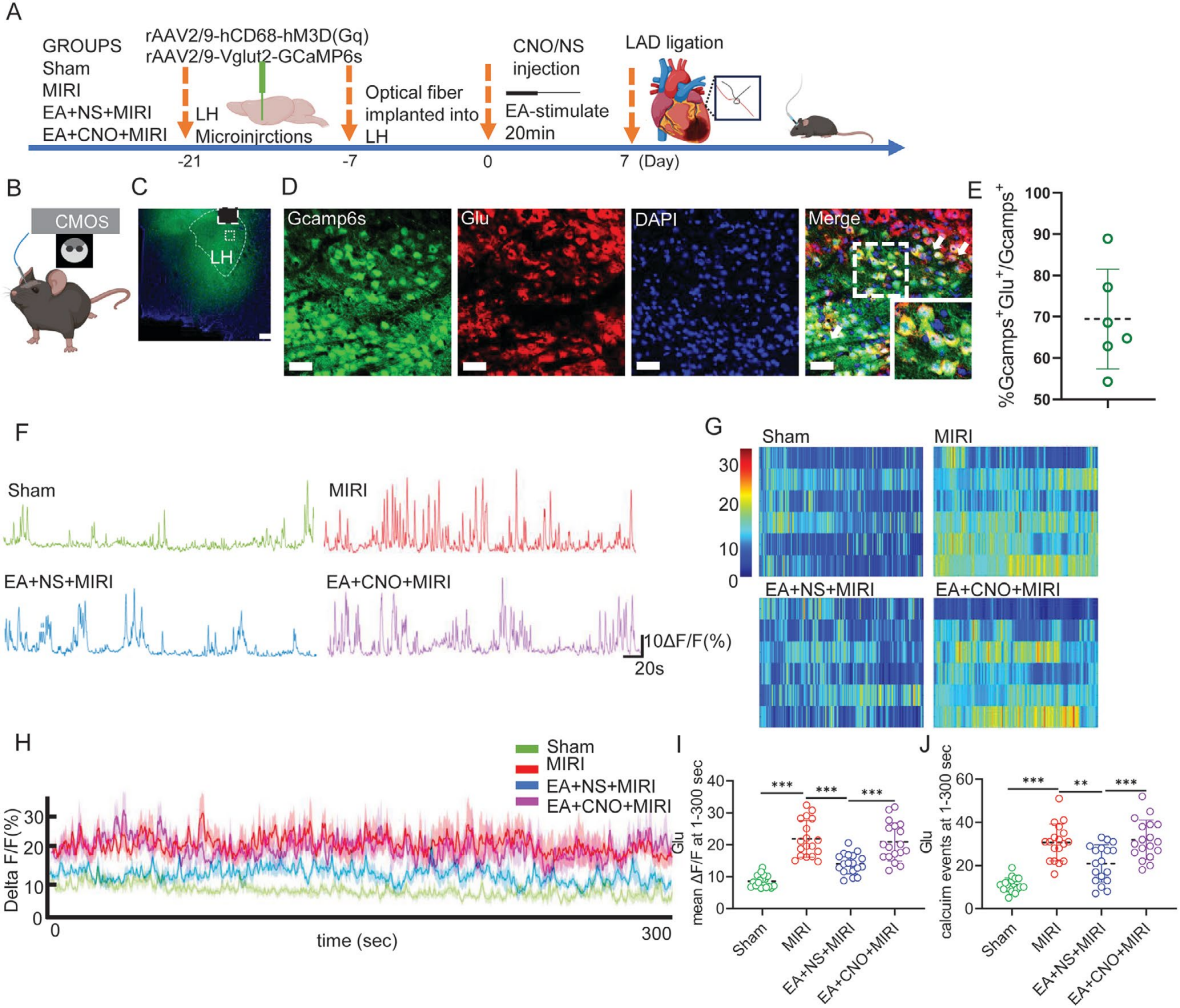
EA‐pre inhibits LH^microglia^ to reduce LH^Glu^ neuronal activity. (A) A strategy diagram for recording calcium signals in LH^Glu^ neurons. (B,C) LH virus injection location. Scale bar = 400 μm. (D) Validated the expression of calcium indicators. (E) Validated the expression efficiency of calcium indicators. (F) Comparison of the original trajectories of calcium activity in LH^Glu^ neurons in 300 s in each group of mice. (G) Comparison of thermograms of Δ*F*/*F* over time trajectories in groups of mice. (H) Comparison of Δ*F*/*F* with time trajectory at 470 nm for each group of mice. (I) The statistical analysis of Δ*F*/*F* for 1–300 s in LH^Glu^ neurons in each group of mice. (J) The statistical analysis of Δ*F*/*F* for 1–300 s calcium events occurring in LH^Glu^ neurons in each group of mice. All data are represented by one‐way ANOVA with Tukey's post‐test, *n* = 6 mice per group. **p < 0.01 and ***p < 0.001.

Further exploration of the effect of microglial engulfment of inhibitory synapses around LH^Glu^ neurons on the activity of LH^Glu^ neurons, we injected calcium indicator GCaMP6s in the LH and recorded it using in vivo fiber photometry recording after viral expression (Figure 5A–C). We also validated the expression efficiency of calcium indicators (Figure 5D,E). The results showed that EA could inhibit Ca^2+^ activity, whereas activation of microglia attenuated the inhibitory effect of EA (Figure 5F). EA‐pre significantly also reduced the ΔF/F signals and calcium events of the LH^Glu^ neurons. In contrast, activation of microglia similarly attenuated the inhibitory effect of EA‐pre, resulting in a significant increase in mean ΔF/F signal and calcium events in LH^Glu^ neurons (Figure 5G–J). The above results indicate that EA‐pre may inhibit microglial engulfment of inhibitory synapses around LH^Glu^ neurons in MIRI mice, thereby suppressing LH^Glu^ neuronal activity.

### 3.5 EA‐Pre Mediates Microglia in LH to Alleviate MIRI

**FIGURE 6:**
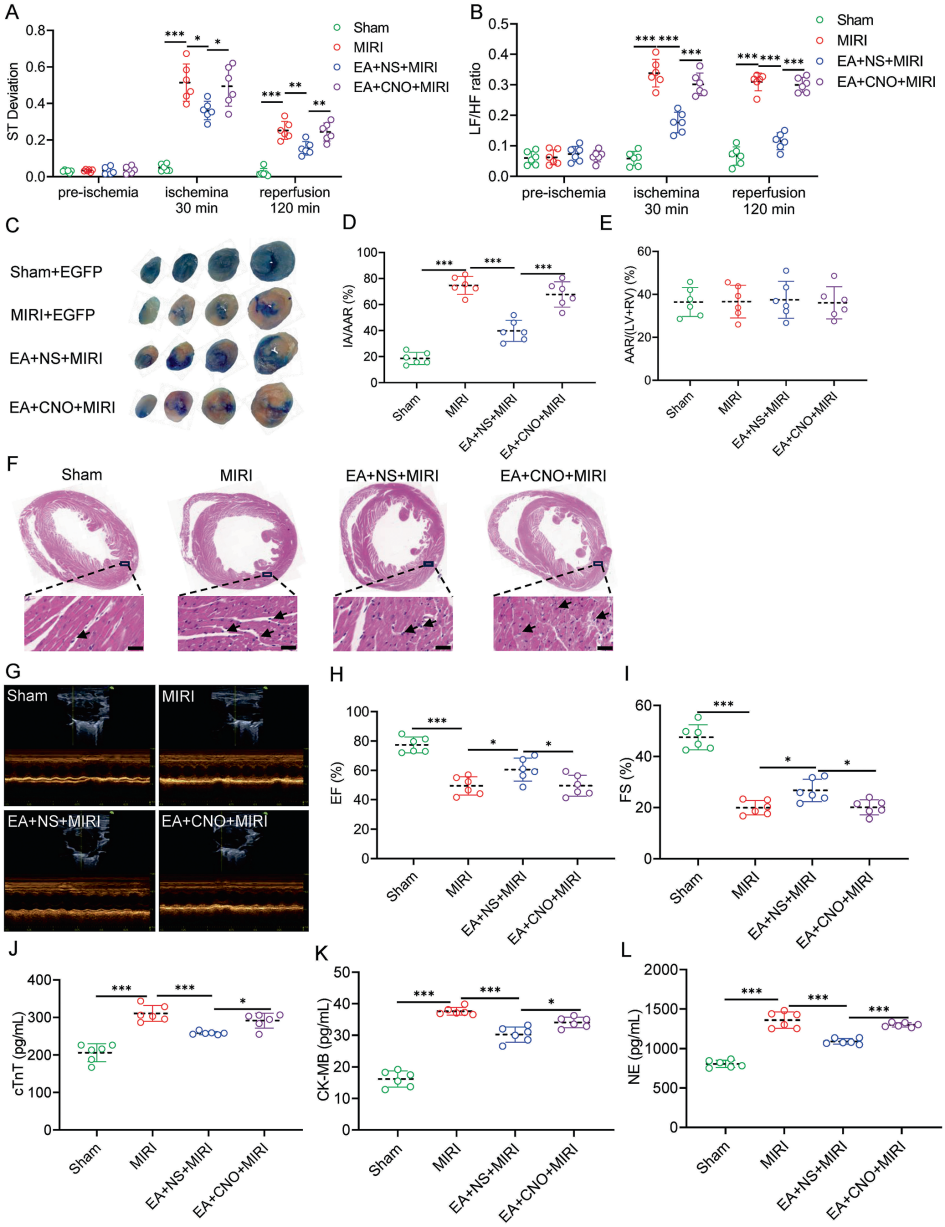
EA‐pre mediates LH^microglia^ to alleviate MIRI. (A) A comparison of S‐T segment deviation in each group of mice. (B) A comparison of the LF/HF ratio in each group. (C) The photos of Evans blue‐TTC double staining. (D,E) A comparison of the percentage of IA and AAR in each group of mice. (F) Hematoxylin and eosin staining showing the morphology of myocardium in each group. Scale bar = 100 μm. (G) Left ventricular echocardiography in each group. (H,I) A comparison of EF and FS values of left ventricular echocardiography in each group. (J–L) The statistical analysis of cTnT, CK‐MB and NE concentrations of left ventricular myocardial tissue homogenates in each group. All data are represented by one‐way ANOVA with Tukey's post‐test, *n* = 6 mice per group. *p < 0.05, **p < 0.01 and ***p < 0.001.

Next, we performed ECG recordings on the mice (Figure S4A). During MIRI, targeted activation of LH^microglia^ weakens the inhibitory effect of EA‐pre on the S‐T segment, exacerbating myocardial ischemia (Figure 6A). The LF/HF ratio also suggested that activation of microglia affects the balance of ANS and increases SNS activity (Figure 6B). Furthermore, we found that the activation of microglia reversed the protective effect of EA on ischemic myocardium and increased IA (Figure 6C,D). Interestingly, there was still no statistically significant difference in AAR among the groups (Figure 6E). Similarly, we found that activation of LH^microglia^ attenuated the therapeutic effect of EA on myocardial tissue injury and infiltration of inflammatory factors (Figure 6F). The activation of LH^microglia^ also weakened the protective effect of EA on cardiac function (Figure 6G–I) exacerbated myocardial injury (Figure 6J,K), and promoted excessive excitation of SNS (Figure 6L). In summary, EA‐pre may inhibit microglial engulfment of inhibitory synapses around LH^Glu^ neurons, thereby suppressing LH^Glu^ neuronal activity and playing a key role in alleviating MIRI.

## 4 Discussion

Microglia, as inherent immune cells in the brain, are involved in the innate immune response of the organism [32], and play an important role in maintaining CNS homeostasis [33, 34]. When microglia transition to a reactive state, their morphology and quantity will change according to different types of stimuli, resulting in significant functional change. It regulates SNS activity and cardiovascular function by releasing various substances such as cytokines, chemokines, and growth factors [35].

MIRI not only induces apoptosis of hippocampal cells [36, 37, 38], but also accompanies neuroinflammatory reactions, and is significantly associated with overactivation of microglia [39]. The pro‐inflammatory microglia in the hypothalamus may affect cardiovascular function through SNS and humoral regulation, leading to heart failure [40, 41, 42, 43]. Inhibition of microglia in the hypothalamus can lead to a decrease in SNS excitability [44]. However, the neuroimmune mechanisms of microglia in EA‐pre mediated alleviation of MIRI remain unclear. Therefore, this study found that during MIRI, LH^microglia^ is stimulated by inflammation and activates engulfment, thereby enhancing the excitability of SNS and exacerbating myocardial injury. EA‐pre inhibited the engulfment by LH^microglia^, weakened the excitability of SNS, and protected cardiac function. The activation of microglia reversed the above situation.

Recent studies have found that microglia are in direct contact with neurons and that microglia are involved in activities that regulate neurogenesis, synaptic plasticity, neuronal firing, and neurodegeneration and regeneration in response to external injury [45]. It and related immune molecules can selectively engulf excitatory and inhibitory synapses to regulate neuronal activity [46]. Our previous research found that LH^Glu^ neurons are important targets for EA‐pre to regulate SNS and alleviate MIRI [23]. In the present study, we applied a combination of in vivo electrophysiological recordings, sparse neuron type‐specific labeling, three‐dimensional reconstruction, and fiber photometry recordings to find that LH^microglia^ may inhibit the activity of LH^Glu^ neurons by engulfing inhibitory synapses around LH^Glu^ neurons and play a key role in EA‐pre mediated alleviation of MIRI. However, the molecular mechanism of its neuroimmune response remains unclear. Next, we will further explore its neuroimmune molecular mechanism through techniques such as single‐cell sequencing [47], spatial transcriptomics [48], metabolomics [49] and patch clamp [50].

## 5 Conclusions

In summary, we confirm that EA‐pre inhibits microglial engulfment of inhibitory synapses around LH^Glu^ neurons in MIRI mice, thereby suppressing LH^Glu^ neuronal activity, reducing SNS output, and ultimately exerting cardioprotective effects. Given the limitations of drug selection in the clinic, this finding raises the possibility of developing a safe and effective targeted bioelectronic therapy based on the EA‐pre approach, which is expected to better serve the clinic.

## Ethics Statement

The animal experiments were approved by the Science and Technology Ethics Committee of the Anhui University of Chinese Medicine (Approval No. AHUCM‐mouse‐2022083). This article did not contain any studies with human participants.

## Conflicts of Interest

The authors declare no conflicts of interest.

## Supporting information


**Figure S1:** EA‐pre effectively protects cardiac function.

**Figure S2:** Microglia regulate the electrical activity of LH neurons during MIRI.

**Figure S3:** Verification of virus injection site in LH.

**Figure S4:** ECG signal recording.

## Data Availability

The data that support the findings of this study are available from the corresponding author upon reasonable request.
