# Supplementary figures and images for: Electroacupuncture Pretreatment Alleviates Myocardial Ischemia–Reperfusion Injury by Inhibiting Engulfment by Microglia in the Lateral Hypothalamus
Source: CNS Neurosci Ther. 2025 Sep 4;31(9):e70595. doi: 10.1111/cns.70595 (PMC12409074; doi:10.1111/cns.70595)

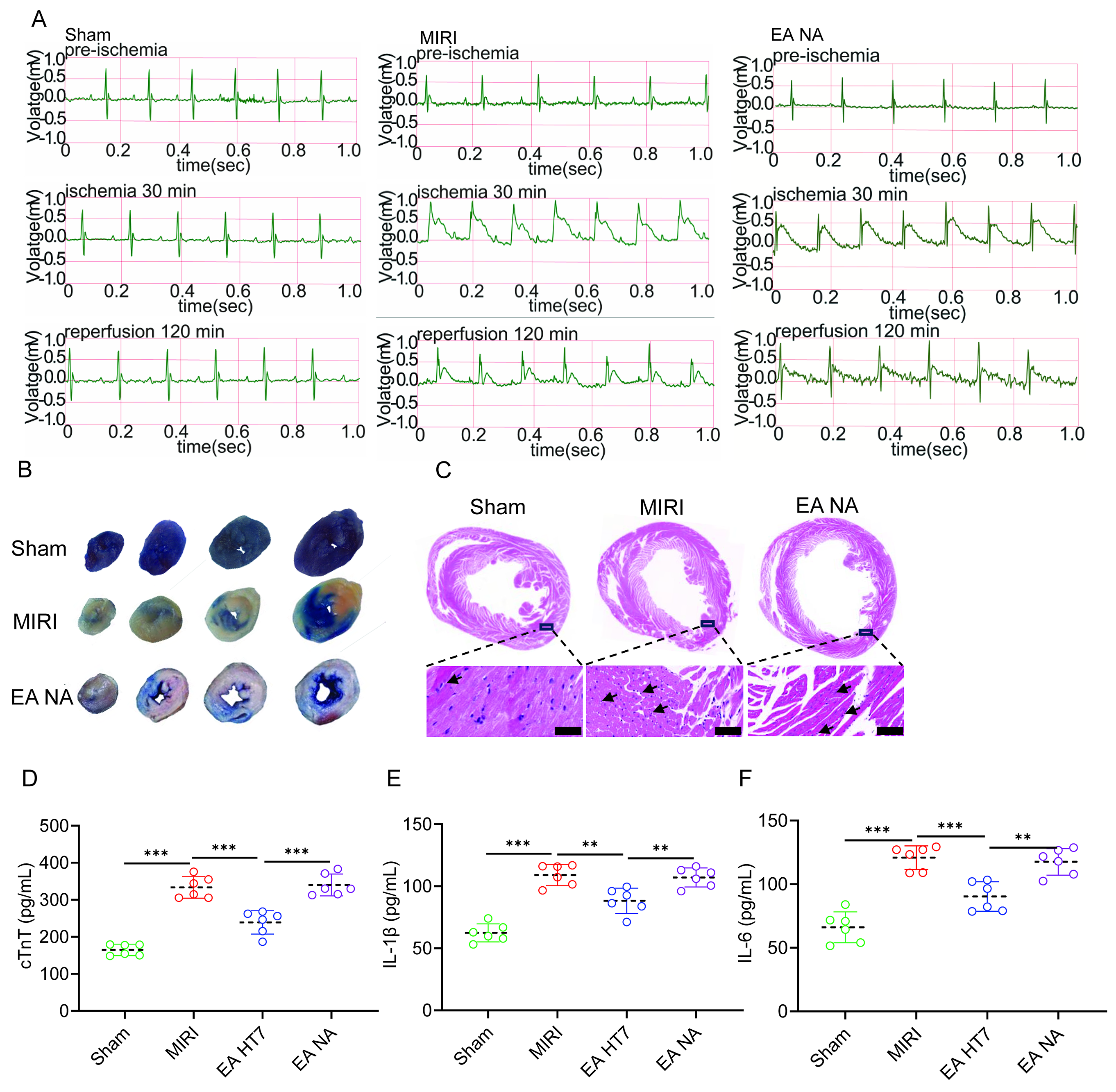

Supplement: Supplementary file 1 — Figure S1: EA‐pre effectively protects cardiac function. [file CNS-31-e70595-s002.tif]

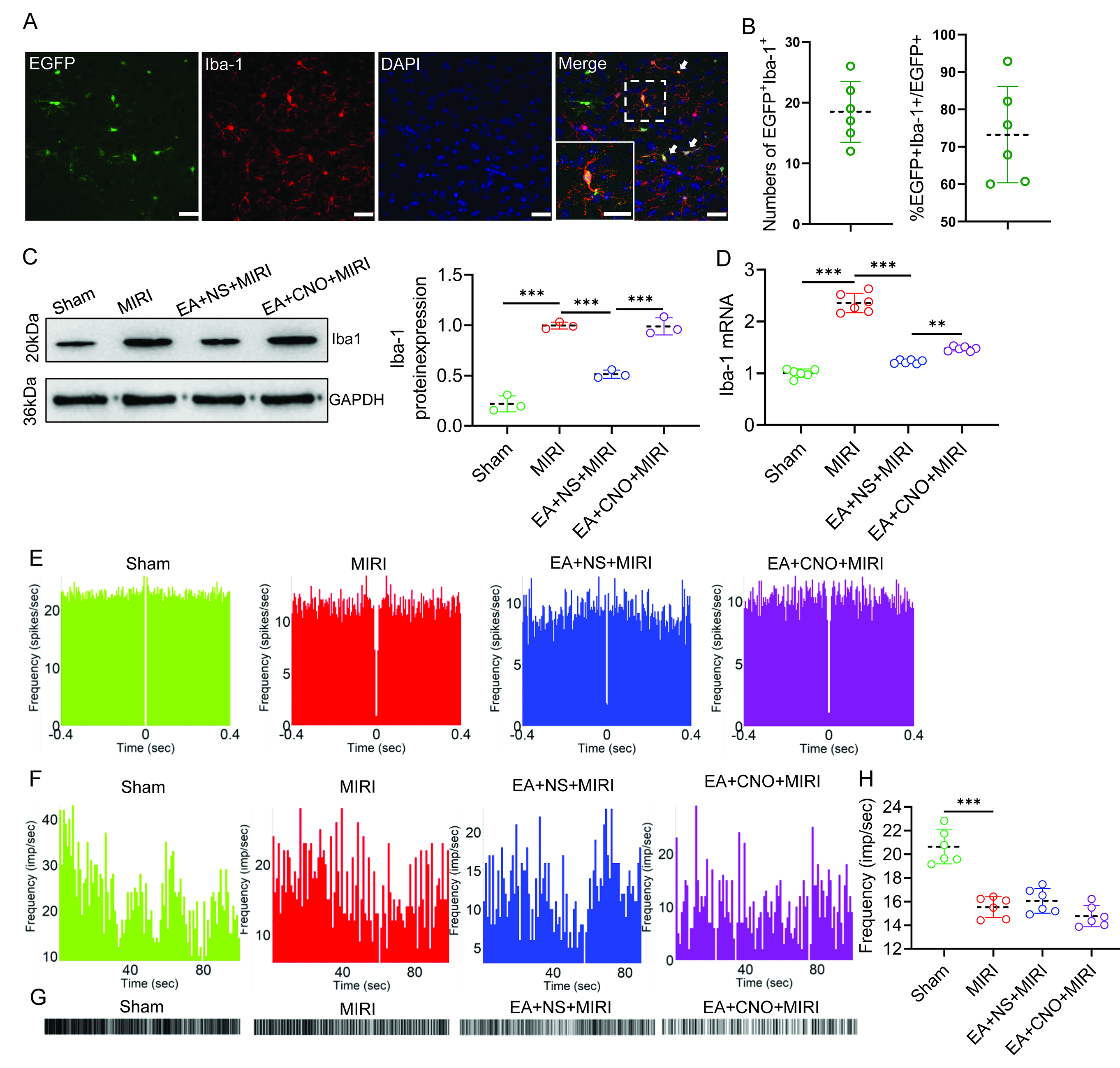

Supplement: Supplementary file 2 — Figure S2: Microglia regulate the electrical activity of LH neurons during MIRI. [file CNS-31-e70595-s001.tif]

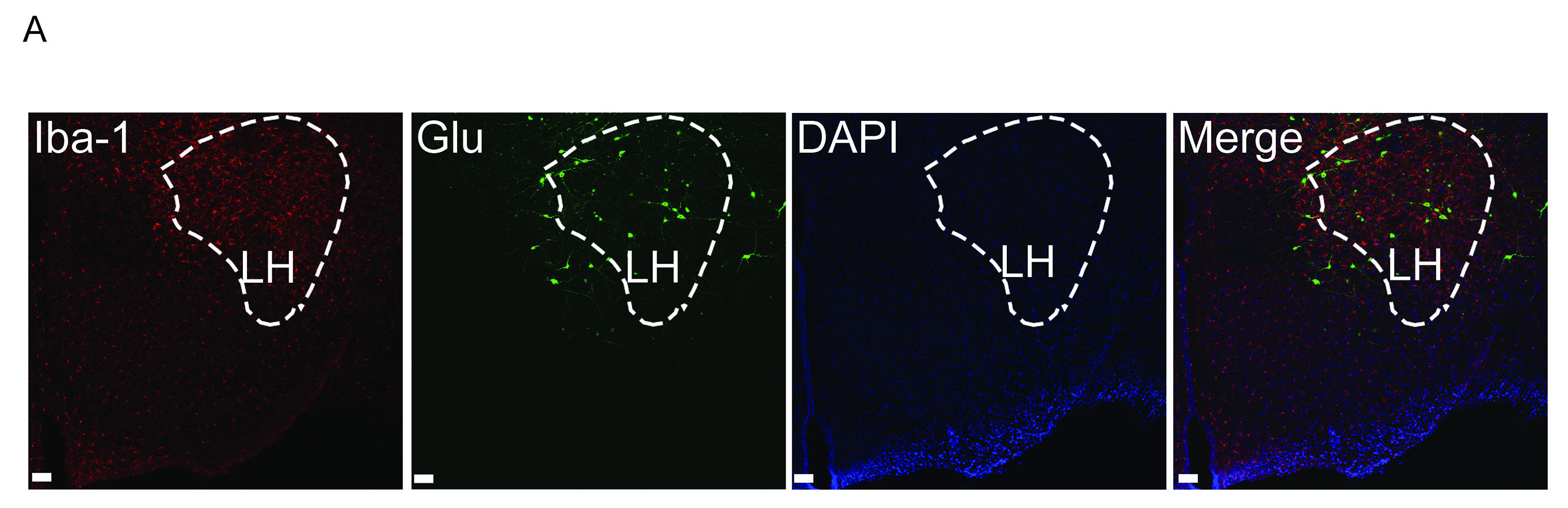

Supplement: Supplementary file 3 — Figure S3: Verification of virus injection site in LH. [file CNS-31-e70595-s003.tif]

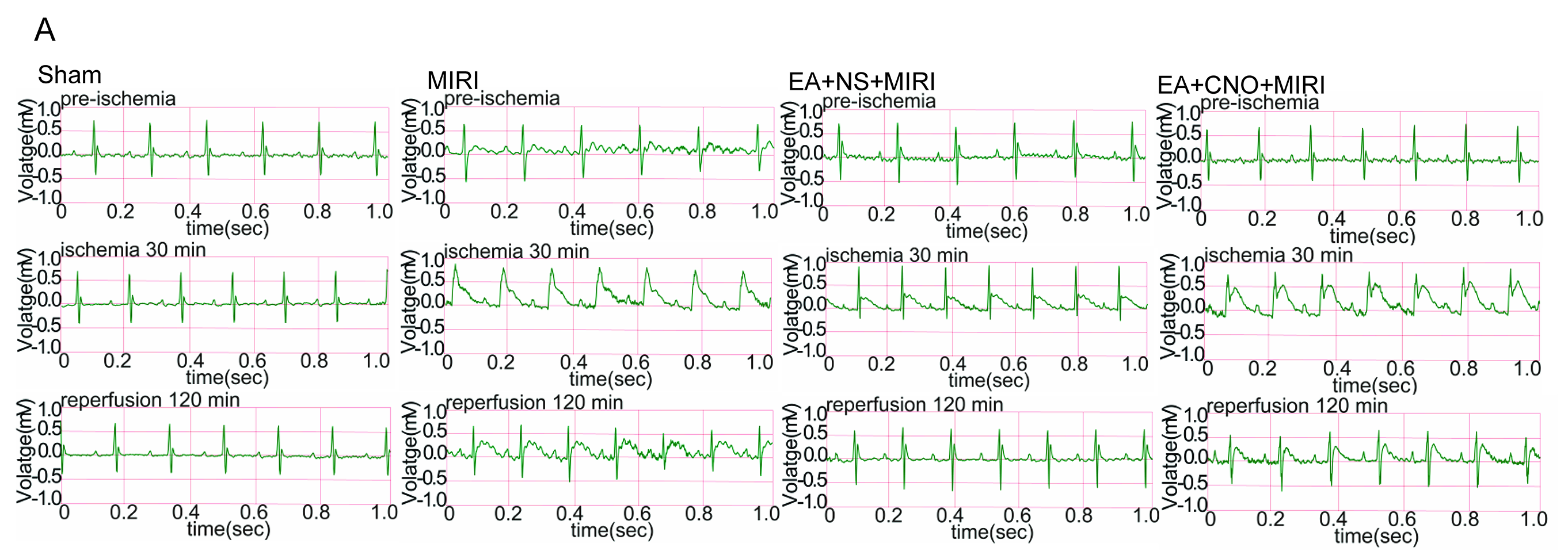

Supplement: Supplementary file 4 — Figure S4: ECG signal recording. [file CNS-31-e70595-s004.tif]
